# Percutaneous MR-guided interventions using an optical Moiré Phase tracking system: Initial results

**DOI:** 10.1371/journal.pone.0205394

**Published:** 2018-10-16

**Authors:** Urte Kägebein, Frank Godenschweger, Brian S. R. Armstrong, Georg Rose, Frank K. Wacker, Oliver Speck, Bennet Hensen

**Affiliations:** 1 Department of Diagnostic and Interventional Radiology, Hannover Medical School, Hannover, Germany; 2 Department Biomedical Magnetic Resonance, Otto-von-Guericke University Magdeburg, Magdeburg, Germany; 3 *STIMULATE* – Solution Centre for Image Guided Local Therapies, Magdeburg, Germany; 4 Department of Electrical Engineering, University of Wisconsin Milwaukee, Milwaukee, Wisconsin, United States of America; 5 Chair in Healthcare Telematics and Medical Engineering, Otto-von-Guericke University Magdeburg, Magdeburg, Germany; Universiteit Twente, NETHERLANDS

## Abstract

The aim of this study was the development and evaluation of a real-time guidance support using optical Moiré Phase Tracking (MPT) for magnetic resonance (MR) guided percutaneous interventions. A gradient echo sequence, capable of real-time position updates by the MPT system, was modified to enable needle guidance based on four rigidly attached MPT markers at the back of a needle. Two perpendicular imaging planes were automatically aligned along the calibrated needle and centered at its tip. For user guidance, additional information about the needle trajectory and the tip to target distance were added as image overlay. Both, images and guiding information were displayed on the in-room monitor to facilitate MR guided interventions. The guidance support was evaluated by four experienced interventional radiologists and four novices targeting rubber O-rings embedded in a custom-made phantom on a 3T wide-bore MRI system (80 punctures). The skin to target time, user error, system error and total error were analyzed. The mean skin to target time was 146s±68s with no statistically significant difference between experts and novices. A low mean user error (0.91mm±0.43mm), system error (0.53mm±0.27mm) and total error (0.99mm±0.47mm) was reached in all directions. No statistically significant difference in user error, system error and total error could be found between experts and novices. The presented tracking and image guidance system combined with the user interface offers continuous and interactive control of the imaging plane while puncturing in the magnet enabling accurate real-time feedback for both, experienced and non-experienced users.

## Introduction

Magnetic resonance imaging (MRI) offers unique approaches to not only diagnose but also treat cancer [1–3]. Compared to CT or ultrasound, MRI has superior soft tissue contrast, lacks ionizing radiation and enables complex double-oblique trajectories [4]. Furthermore, MRI has the unique ability to acquire functional information (e.g. temperature, perfusion). This can be used to accurately control the success of thermal ablation therapies [4]. However, interventional MRI (iMRI) is still limited to specialized clinical centers due to access and workflow limitations [5, 6]. In order to make MRI guidance a standard procedure, the workflow has to be adjusted to be more time efficient. This is especially important for the so called freehand technique [2, 4]. Here an interventional device is advanced to the target using continuous imaging. A dedicated interventional platform, such as the Interactive Front End (IFE, Siemens Healthineers, Erlangen, Germany) makes the procedure more intuitive [2]. In contrast to classical in-out procedures, real-time imaging allows for compensation of organ movement [7]. Main disadvantages include the need for manual slice adjustment, in particular when the needle or the target is lost from the imaging plane. This process can quickly become cumbersome and time-consuming without a well-coordinated team, especially in cases of complex trajectories [2]. In this realm, needle guidance support and streamlined interfaces might be able to improve precision and procedure time.

Several attempts have been made to develop an appropriately interactive guidance support [8]. MR-compatible robotic assistance systems have been presented. However, the in-bore approaches do not provide haptic feedback [9], whereas the out-of-bore applications miss real-time imaging feedback that is essential for moving organs such as the liver [10]. The same applies for an out-of-bore augmented reality system [11, 12]. Other tracking techniques utilize detection of the needle artifact or additional passive markers. The detection algorithms, however, are considered susceptible to errors in case of fast motions and image distortions [13]. Active optical tracking systems are a more common modality for intra-operative instrument tracking. Typically, a stereo-vision camera system detects the position of several light emitting diodes or retro-reflective markers attached to the back of the instrument. Non MRI-compatible camera systems need to be positioned in a safe distance to the MRI and thus instrument navigation inside the bore is strongly affected by line-of-sight limitations. Therefore, such systems have only been exploited for out-of-bore approaches or in combination with open MR systems [14, 15], which are no longer available. Therefore, appropriate interactive in-bore guidance methods are still lacking.

This paper presents a new tracking system and sequence that can be completely integrated into the standard clinical interface of an MRI scanner. We use a precise MRI-compatible in-bore optical Moiré Phase tracking system, which facilitates interactive control of the imaging slices during freehand puncture inside the magnet. Real-time needle guidance performance was evaluated regarding targeting error and skin to target time by experienced interventional radiologists and novice users.

## Material and methods

### Hardware setup

**Fig 1:**
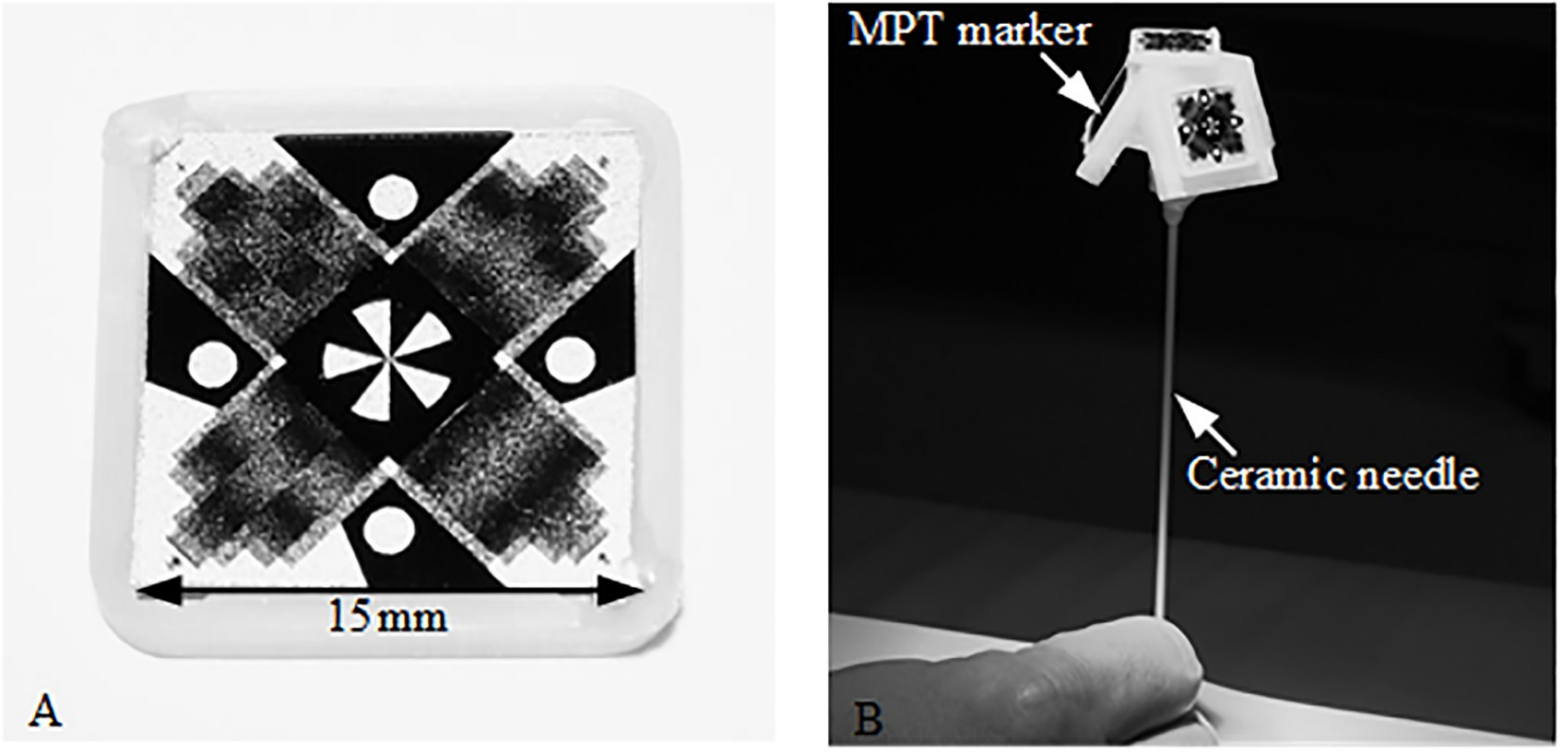
Optical MPT system with interventional tool for real-time guidance. (A) The MR-compatible tracking camera detects the position and orientation of a planar MPT marker shown in. (B) For real time needle guidance four MPT markers were rigidly attached to a ceramic needle with a dedicated holding device.

Optical MPT technology (Metria Innovation Inc., Milwaukee, United States) was used for real-time needle guidance. It consists of a planar MPT marker (15mm x 15mm; see Fig 1A), a MR-compatible tracking camera with a single lens and fixed focus (exposure time 200μs), mounted inside the magnet bore close to the isocenter, and a tracking computer, as already described in detail previously [16, 17]. The MPT technology allows for high precession six degrees of freedom (DOF) tracking (rotation ~0.01°, translation ~10μm) by estimating the changes of the moiré patterns as well as of additional landmarks on the MPT marker [16]. The system was originally developed for prospective motion correction of MR brain images with an isotropic resolution of up to 250μm at 7T [18]. This application requires only a small tracking field of view of ~91mm x 64mm at a MPT marker distance to the camera of ~187mm (Note: The available field of view depends on the MPT marker distance to the camera as well as their orientation to each other. The present values are given for a parallel orientation of the MPT marker to the camera.). The working volume of this system is very small and optimized for correction of head motion. For the application in interventional procedures, the tracking camera system was adapted in cooperation with the manufacturer for a larger tracking field of view (~158mm x 128mm at a MPT marker distance towards the camera of ~182mm), allowing more flexibility in camera, patient and tool positioning for interventional procedures. In addition, the optical system was modified to use infrared illumination.

A handpiece containing four MPT markers was designed, 3D printed and attached to a cylindrical ceramic needle prototype (12G/2mm diameter, 150mm length) with a single-sided tip (see Fig 1B). A ceramic cylinder was used instead of a commercially available metallic biopsy needle to reduce the needle artifact. This study focuses on error evaluation and only a ceramic needle without related local signal dropouts guarantees an accurate validation of the final needle position from high resolution MRI. In contrast to metallic instruments, the needle appearance in the image represents mainly the lack of water protons. Thus, it can be assumed that the center of the signal void corresponds to the center of the ceramic needle. The tip position and orientation of the ceramic needle relative to the attached MPT markers was determined using an in-house calibration device and procedure with an additional MPT marker.

### Software setup

**Fig 2:**
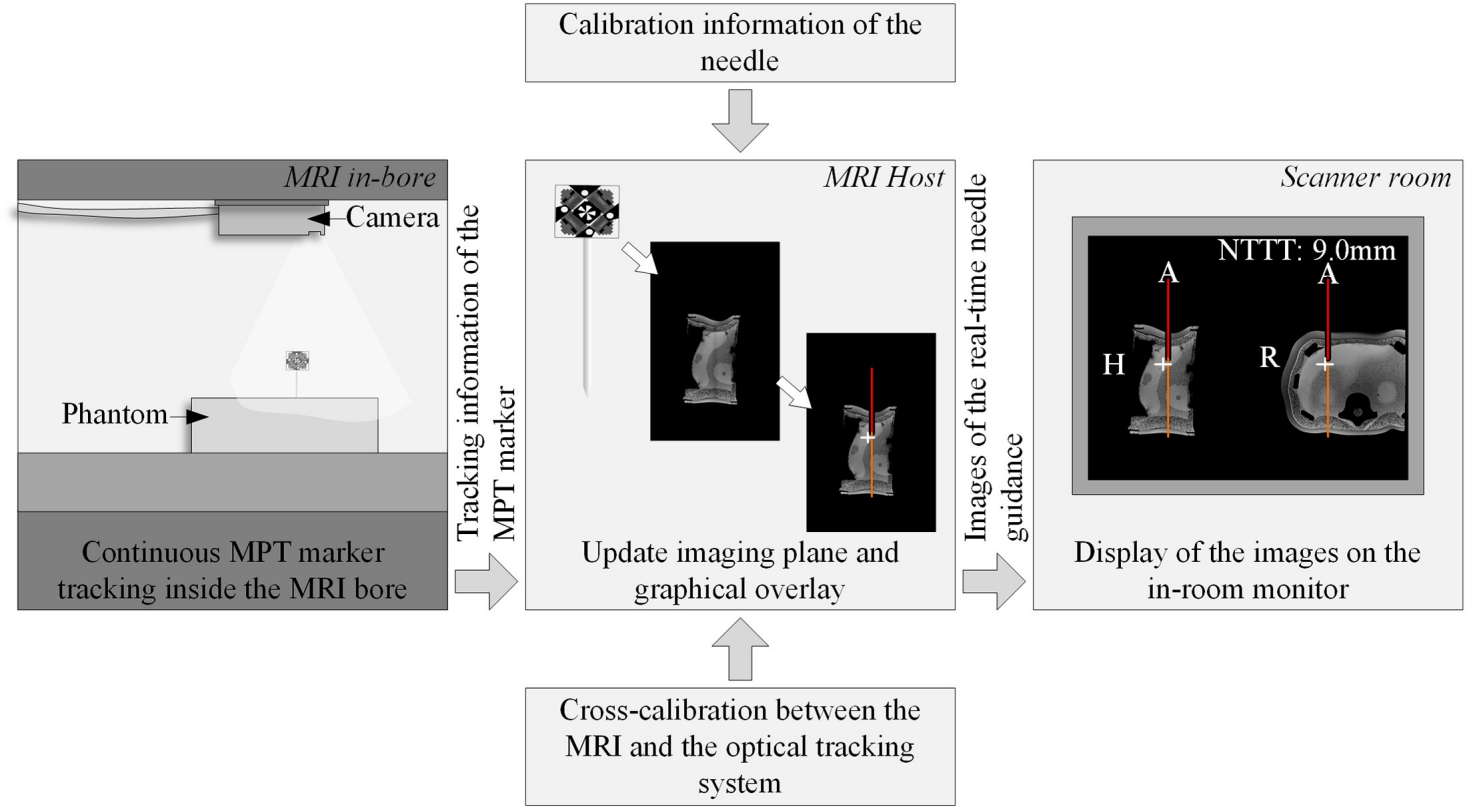
Overview of the software implementation. The real-time position of the MPT marker was continuously sent to the MRI Host in the control room. Based on it, the modified GRE sequence automatically aligned the imaging planes along the needle further utilizing the calibration information of the needle as well as the cross-calibration between the MRI and the optical tracking system. Additionally, a graphical overlay was produced. The resulting images of the needle guidance were sent in real-time towards the in-room monitor in the scanner room.

A gradient echo (GRE) sequence, capable of real-time position updates according to the pose information from the MPT system for motion correction [16], was modified for real-time needle guidance within the Integrated Development Environment for Applications and Image Calculation Environment (Siemens, Germany). The tracking information of the MPT marker was sent in real-time (9fps) from the MPT tracking computer to the MRI Host within UDP packets via an Ethernet connection. The modified GRE sequence translated the latest real-time position of the MPT marker into MRI coordinates utilizing the cross-calibration and automatically aligned two imaging planes along the orientation of the needle and centered at the needle tip based on the calibration information (see Fig 2). The imaging planes were perpendicular to each other and aligned most closely to the standard axial, coronal or sagittal plane for best image recognition and interpretation.

**Fig 3:**
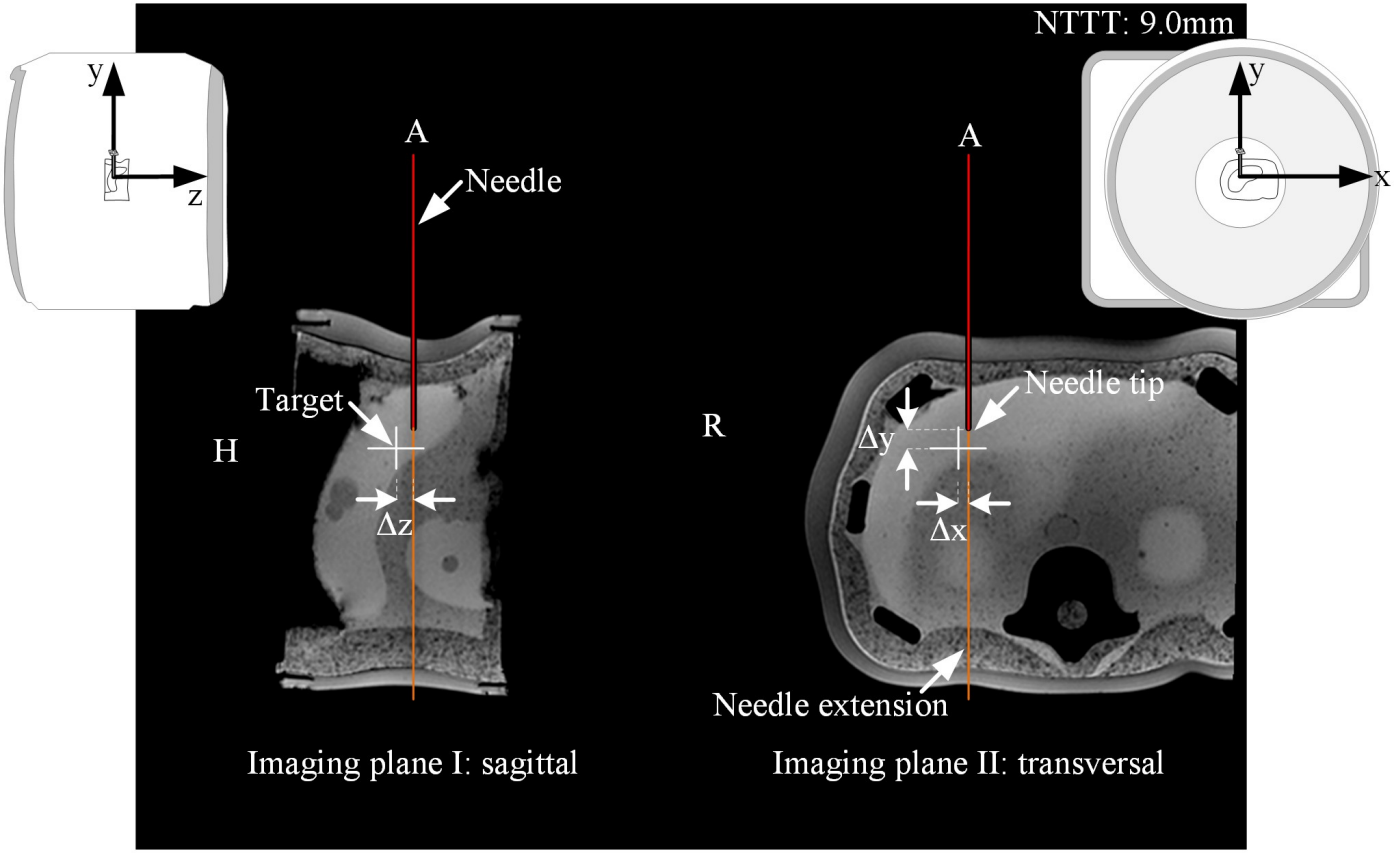
Schematic drawing of the real-time needle guidance with the MPT system. Two perpendicular imaging planes were aligned along the needle trajectory and centered at the needle tip. The additional graphical overlay contains information about the 3D distance $d_{3D}\;=\;\sqrt{\Delta x^{2}+\Delta y^{2}+\Delta z^{2}}$ between the needle tip and the predefined target as well as the 2D distance of $d_{2D}\;=\;\sqrt{\Delta x^{2}+\Delta z^{2}}$.

The tracking information was further utilized to visualize the position of the needle and its extension (red and orange line, see Fig 3), whereby the end of the red line marked the needle tip ***P***_TipTracked_, as tracked by the MPT system. For further user guidance, the position of a predefined target ***P***_Target_ was added as a colored cross. The color of the cross depended on the 3D distance *d*_3*D*_ between the needle tip and the target (see Fig 3): (1) white for *d*_3*D*_ > 5mm; (2) orange for 3mm < *d*_3*D*_ ≤ 5mm; (3) yellow for 1mm < *d*_3*D*_ ≤ 3mm and (4) green for *d*_3*D*_ ≤ 1mm. The 2D distance *d*_2*D*_, indicating trajectory deviations from the planned needle path, was coded using color changes of the needle extension. In addition, information about the remaining puncture track Δ*y* from the needle tip to the target was added as text overlay ‘NTTT: 20.0mm’ (needle tip to target (NTTT), see Fig 3). Both imaging planes together with the graphical overlay were continuously updated and displayed side-by-side on the real-time reconstruction display of the MRI. Those images were additionally sent to the in-room monitor within the scanner room to provide a constant feedback of the real-time needle guidance.

### Hands-on user study

The proposed real-time needle guidance was evaluated by four experienced (radiologists, interventional experience: 10.75years±5.68years) and four non-experienced users (engineers and physicists, no interventional experience) in order to assess targeting error and skin to target time. The users were recruited based on their experiences (participant recruitment date: August until September 2017). The number of subjects was not based on a power analysis due to missing hypotheses on the effect size and inter-subject variance. It was rather chosen to be most reasonable within the resource and time restrictions. Each user was asked to hit different predefined targets within a phantom using the MPT system. In total, ten needle insertions were performed by each user resulting in an absolute sample size of eighty. Prior to the experiments, each user was allowed to conduct one needle insertion for training purposes (~6.5min).

#### Installation procedure

**Fig 4:**
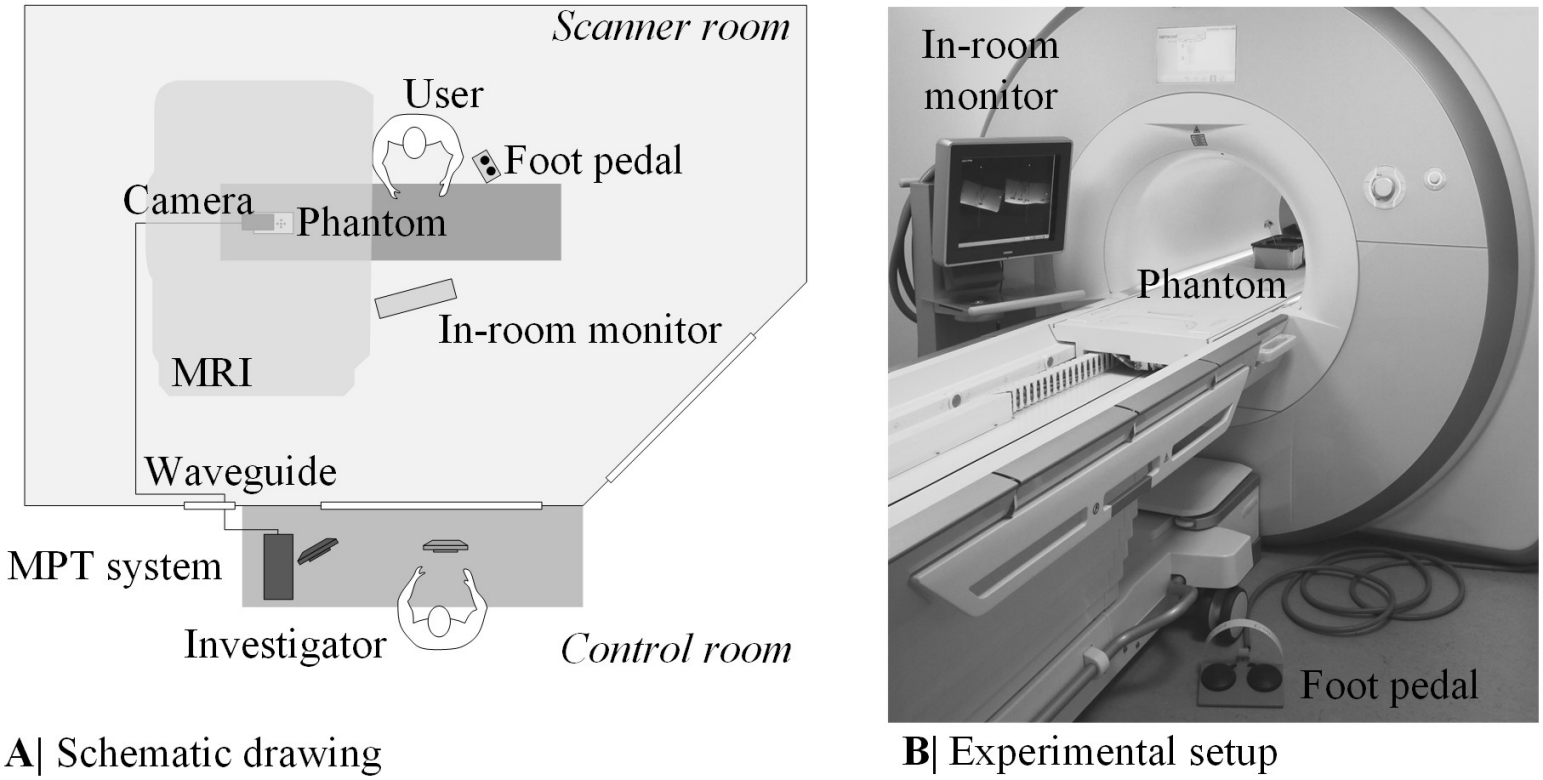
Experimental setup of the user study. A schematic drawing of the experimental setup is given in (A). The image (B) shows the real setup during the hands-on user study.

The experiments were performed on a wide-bore 3T MRI (Magnetom Skyra, Siemens, Germany) using three elements of the spine coil (see Fig 4). The camera was mounted inside the magnet bore close to the isocenter using Velcro. After an additional pre-heating phase of 100min, a cross-calibration between the MRI coordinate system and the camera coordinate system was performed. Further information regarding the cross-calibration can be found under *Stucht et al*. [17]. This step was necessary to obtain the real-time MPT marker pose with respect to the MRI coordinate system. The biopsy phantom was filled with jelly candle wax and ten rubber O-rings mimicking target lesions (9mm inner diameter, 1.5mm height). An opaque layer covered the phantom.

#### Real time needle-guidance

Prior to the start of the experiments, the entry and target points were defined by the investigator in the control room using the software IFE and a T1-weighted planning dataset (3D GRE, acquisition time (TA) = 11s, TE/TR = 1.57ms/4.06ms, resolution 2.15mmx1.61mmx2.56mm). The target point ***P***_Target_ was set within the rubber O-rings. After briefly looking at the planned trajectory, the first step was, to determine the entry point using the laser crosshair of the MRI and an MR-compatible ruler as previously described in *Rothgang et al*. [2]. In the second step of the procedure the user advanced the needle towards the predefined target while simultaneously controlling the slice position with the implemented real-time needle guidance (2D GRE, TA = 1s, TE/TR = 3.76ms/8.0ms, resolution 2.08mmx2.08mmx5mm). The two imaging planes and the graphical overlay were continuously updated and displayed on the in-room monitor located next to the magnet (Fig 4). In the beginning of the targeting procedure, the users were instructed to adjust the needle orientation to the planned trajectory until the color of the needle extension turned green, indicating that the actual needle trajectory and the planned trajectory are smaller than 1mm. After that they firmly inserted the needle along the planned trajectory controlling the needle depth with the text overlay NTTT and the color of the cross. Once the cross turned green and NTTT came close to 0mm, the users stopped the real-time needle guidance with the foot pedal. To assess the remaining targeting error, a high-resolution 3D validation dataset automatically aligned to the needle trajectory centered at the final needle tip ***P***_TipTracked_ was acquired (3D GRE, TA = 114s, TE/TR = 5.91ms/30ms, resolution 0.5mm isotropic, including 3D distortion correction). The dataset was used to measure the tip of the visible ceramic needle artifact (true needle tip position ***P***_TipTrue_).

#### Error evaluation

The skin to target time ***t***_SkinToTarget_ was recorded from starting the real-time needle guidance until pressing the foot pedal. The targeting error was subdivided into three different errors in x, y and z dimension. The user error was calculated as the distance between the final tracked needle tip ***P***_TipTracked_ and the predefined target ***P***_Target_. It shows how well the user exploited the graphical overlay of the real-time needle guidance. The system error is the difference between the final tracked needle tip ***P***_TipTracked_ and the true needle tip position ***P***_TipTrue_. It is related to the accuracy of the cross-calibration between the MRI and the optical tracking system as well as the needle calibration. Thus it reflects how well the information about the tracked needle is exploited to automatically align the image plane along the needle. The total error was calculated comparing the true needle tip position ***P***_TipTrue_ with the predefined target ***P***_Target_. A two-sample t-test of equal means for unpaired data was used to analyze differences in skin to target time as well as mean user error, mean system error and mean total error for experienced and non-experienced users (significance level α = 0.05). The total error in x, y and z direction of all users was examined to detect a possible preferred error direction with the aid of a repeated measures analysis of variance. Due to the small group size, inter- and intra-group variations were not separated.

## Results

**Fig 5:**
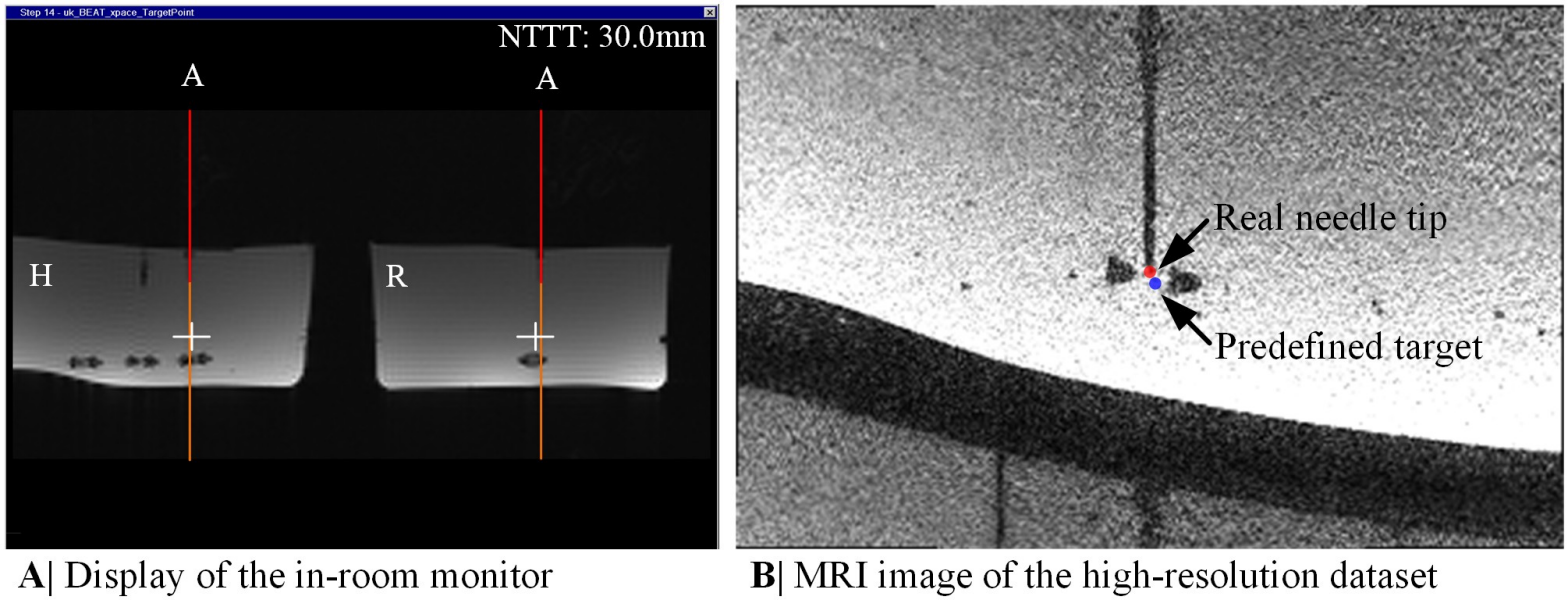
Real-time needle guidance with the MPT system during the user study. (A) The display of the in-room monitor presents images of the real-time needle guidance sequence during the user study together with its graphical overlay. (B) The present MRI image was extracted from one high-resolution dataset. The colored dots mark the detected real needle tip position (red dot) and the predefined target (blue dot).

Once the real-time needle guidance sequence was started, the modified GRE sequence automatically aligned the two imaging planes along the needle centered at the needle tip using the pose information of the MPT marker. The images were sent in real-time with negligible displaying delay to the in-room monitor providing a constant feedback for the users about the needle trajectory, needle extension and target-tip distance (see Fig 5A). Thus, the users were able to continuously navigate the needle to the predefined targets by translating the needle within the MRI bore and utilizing the real-time feedback on the in-room monitor. The update rate of the real-time needle guidance including the processing time for image plane update amounted to 1fps, being equivalent to the acquisition time of the GRE-sequence. The update rate of the MPT system (including internal system delays) was much higher (9fps), but the GRE-sequence utilized only the latest pose information of the MPT marker. In total, the mean skin to target time was 146s±68s for the experts and 165s±55s for the novices (see Table 1). There was no significant difference between experienced and non-experienced users (p = 0.17).

**Table 1:** Results of the hands-on user study with the MPT system.

		Experts (n^a^ = 40)	Novices (n = 40)	All paths (n = 80)
**User error (mm)**	Δx Δy Δz Mean 3D	0.72 (±0,56) 1.08 (±0.88) 0.90 (±0.66) 0.90 (±0.39) 1.81 (±0.85)	0.86 (±0.68) 0.97 (±0.94) 0.96 (±0.61) 0.93 (±0.46) 1.83 (±0.96)	0.79 (±0.62) 1.02 (±0.91) 0.93 (±0.63) 0.91 (±0.43) 1.82 (±0.90)
**System error (mm)**	Δx Δy Δz Mean 3D	0.74 (±0.50) 0.37 (±0.29) 0.56 (±0.40) 0.55 (±0.27) 1.12 (±0.49)	0.59 (±0.49) 0.38 (±0.29) 0.55 (±0.50) 0.50 (±0.27) 1.03 (±0.52)	0.66 (±0.50) 0.37 (±0.29) 0.55 (±0.45) 0.53 (±0.27) 1.07 (±0.50)
**Total error (mm)**	Δx Δy Δz Mean 3D	1.06 (±0.80) 1.00 (±0.78) 0.89 (±0.70) 0.99 (±0.45) 1.96 (±0.88)	1.08 (±0.77) 0.96 (±0.98) 0.98 (±0.88) 1.00 (±0.51) 2.04 (±1.07)	1.07 (±0.78) 0.98 (±0.88) 0.93 (±0.79) 0.99 (±0.47) 2.00 (±0.97)
**Skin to target time (s)**		146 (±68)	165 (±55)	155 (±62)

An overview of the user error, system error and total error as well as the skin to target time for experts, novices and all paths are presented.

^a^ Sample size.

Using the high-resolution validation dataset, the needle tip positions could be successfully determined in all cases (see Fig 5B). All users punctured the predefined targets with a mean user error of 0.91mm±0.43mm with a slightly but not significant lower mean user error for experts (0.90mm±0.39mm) than for novices (0.93mm±0.46mm; see Table 1; p = 0.75). The respective mean system error was 0.53mm±0.27mm. The mean total error for experts and novices amounted to 0.99mm±0.45mm and 1.00mm±0.51mm, respectively. Comparing the experts with the novices, no significant differences in the mean system error (p = 0.41) and mean total error (p = 0.93) were identified. There was no preferred error direction in one of the three coordinates x, y or z (repeated measure analysis of variance: F = 1.0; *df* = 79, 2; p = 0.36).

## Discussion

We have developed and evaluated a system for MRI-guidance support for percutaneous punctures. The MPT system offered continuous and interactive control of the imaging slices in real-time directly from the inside of the MRI magnet while maintaining the freehand convenience. Using two orthogonal imaging planes in combination with a graphical overlay, both, experts and novices have successfully navigated the needle to a predefined target with no significant difference in targeting error or skin to target time. Thus, we conclude, that the developed guidance support seems to be so simple and intuitive that even novices can achieve similar results as experts in terms of speed and accuracy.

Comparing the mean total error in all directions (0.99mm±0.47mm) to other publications, the presented in-bore technique offers higher accuracy and exact instrument positioning. Using the IFE in a similar wide-bore system, a complete out-of-bore technique with an augmented reality system, a passive in-bore marker tracking and an optical tracking system for an in-out technique, *Rothgang et al*., *Wacker et al*., *Oliveira et al*. and *Busse et al*. reported a mean error of 1.8mm±1.5mm, 1.1mm±0.5mm, 1.5mm±1.1mm and 3.9mm±2.4mm, respectively [2, 11, 13, 14]. Most of these studies used MRI compatible metal needles, which might make exact needle verification somewhat difficult. We utilized a ceramic cylinder instead for a more accurate validation of the final needle tip position.

Even with the high precision of the MPT system, further reduction of both, user and system error are desirable because breathing motion and movement will add to the total error in patients [11]. Comparing the mean user error of 0.91mm (±0.43mm) to the mean system error of 0.53mm (±0.27mm), the human factor of the users influenced the total error more than technical limitations. The user error may be improved by a more sensitive color gradation of the cross as well as the needle extension. A threshold of 0.5mm instead of 1mm might allow the interventionalist to bring the needle tip closer to the target. The system error, already relatively small, might benefit from improved calibration. However, it has to be kept in mind, that the error introduced by needle bending cannot be easily compensated. Furthermore, the current study evaluated only a limited number of representative needle positions and orientations relative to the MPT camera or the MRI system. Especially at the outer boundary of the MR imaging volume, the system error will potentially be larger due to magnetic field inhomogeneities or gradient non-linearities if these are not considered. Given the errors reported in the studies mentioned earlier, however, our system seems as it is seems to be ready for clinical use.

This is also true for the skin to target time which was slightly higher (155s±62s) when compared to *Rothgang et al*. (100s±50s). This can be explained by the lower acquisition frame rate of the applied GRE sequence (1fps vs. 2fps) rather than the update rate of the tracking system (9fps) [2]. Therefore, we will try to implement an accelerated sequence with our tracking system. Still, the combination of high accuracy and acceptable skin to target time, even with the current system, makes the MPT system an attractive tool not only for experts but also for less-trained interventional radiologists, who benefit from the intuitive guidance interface.

The MPT tracking camera was mounted inside the magnet bore. In comparison to other optical tracking systems presented for closed bore environments [14], this has many benefits. First, the procedure can be performed within the magnet. Second, line of sight problems were rarely observed in this study. Third, the needle guidance is completely integrated into the standard clinical interface. Although an extra tracking hardware is needed, it can be transferred to any other imaging sequence and MRI model.

As the position of the predefined target within the graphical overlay cannot follow motion, the MPT system may be particularly helpful in non-moving organs such as the prostate in order to treat small lesions close to risk structures. Additionally, musculoskeletal or cerebral interventions are conceivable. However, due to the simultaneous real-time imaging feedback, moving organs such as the liver, kidney or spleen will also benefit from the intuitive guidance support. The interventional radiologist can view the moving lesion and can use the graphical overlay not only for targeting but also to assess the breathing cycle of the patient, which helps to correct the needle path and choose the time for advancing the needle. Previous studies in X-ray angiography and MR-angio overlay have confirmed the usefulness of this approach [19, 6]. In addition, the slice plane adjustment controlled by the interventional instrument simplifies the interaction with the MRI. The MPT system also helps to define the optimal entry point and needle path quite intuitively while the needle is still in the subcutaneous tissue as evidenced in our experimental setup.

One shortcoming of this technical report is the lack of patient data. Although a phantom study represents the optimal way to investigate the basic principle, the skin to target time and to quantify the targeting errors, the MPT based real-time needle guidance needs to be clinically evaluated particularly regarding potential line-of-sight challenges with a patient inside the bore and depending on the needle trajectory. Nonetheless, given that the MPT tracking approach is new, the technical evaluation is necessary. In addition, we only used a GRE sequence which was sufficient for the phantom experiments. Further sequences such as T2 weighted turbo spin echo for enhanced lesion detection as well as needle artifact suppression and balanced steady-state free precession for higher imaging frame rate will be integrated in the future [6]. Besides, the MPT system will be compared to standard procedures (e.g. freehand technique) in a more realistic clinical scenario. Unlike the present study, which was mainly focused on spatial accuracy and procedure time, this future user study will enable the detailed evaluation of the individual user experiences for different tasks using e.g. the NASA Task Load [20] or Likert scales.

In conclusion our phantom study shows that the MPT tracking system presented helps to make a potentially cumbersome process such as MRI-guided percutaneous puncture a real-time procedure. The independence from the experience of the user suggests that the system is intuitive and easy to use. The interventional radiologist is able to control the entire procedure and the real-time imaging next to a patient inside the magnet with the hands on the interventional device only. The proposed approach has the potential to facilitate iMRI-based interventions in the future.

## Supporting information

S1 Movie Video of the in-room monitor presenting images of the real-time needle guidance with its graphical overlay for one subject. (MP4)

S1 Table User error, system error and total error for the individual subjects of the hands-on user study. (XLSX)
